# Shack–Hartmann wavefront sensors based on 2D refractive lens arrays and super-resolution multi-contrast X-ray imaging

**DOI:** 10.1107/S1600577520002830

**Published:** 2020-04-22

**Authors:** Andrey Mikhaylov, Stefan Reich, Margarita Zakharova, Vitor Vlnieska, Roman Laptev, Anton Plech, Danays Kunka

**Affiliations:** a Institute of Microstructure Technology, Karlsruhe Institute of Technology, Hermann-von-Helmholtz-Platz 1, Eggenstein-Leopoldshafen, Baden-Wuerttemberg 76334, Germany; b School of Nuclear Science and Engineering, National Research Tomsk Polytechnic University, 30 Lenin Avenue, Tomsk, Tomsk oblast 634034, Russian Federation; c Institute for Photon Science and Synchrotron Radiation, Karlsruhe Institute of Technology, Hermann-von-Helmholtz-Platz 1, Eggenstein-Leopoldshafen, Baden-Wuerttemberg 76344, Germany

**Keywords:** Shack–Hartmann sensor for hard X-rays, sample-shift multi-contrast imaging, interleaving measurement, phase-contrast imaging, polymer refractive lenses

## Abstract

Different approaches of 2D lens arrays as Shack–Hartmann sensors for hard X-rays are compared. For the first time, a combination of Shack–Hartmann sensors for hard X-rays (SHSX) with a super-resolution imaging approach to perform multi-contrast imaging is demonstrated. A diamond lens is employed as a well known test object. The interleaving approach has great potential to overcome the 2D lens array limitation given by the two-photon polymerization lithography. Finally, the radiation damage induced by continuous exposure of an SHSX prototype with a white beam was studied showing a good performance of several hours. The shape modification and influence in the final image quality are presented.

## Abstract 2

Super-resolution multi-contrast X-ray imaging performed using a Shack–Hartmann sensor for hard X-rays is presented.

## 1. Introduction   

Over the years, sensing wavefront deformations after passing through an object has become of great importance in imaging for many medical and industrial applications. In this way, several multi-contrast imaging techniques using hard X-rays have emerged in recent decades employing synchrotron and laboratory sources (Wilkins *et al.*, 2014 ▸). An example of X-ray imaging methods at the forefront of technology is phase-contrast X-ray imaging using one, two or three optical components between the source and the detector. Among the techniques developed for this purpose are those based on interference (Momose, 2003 ▸; Pfeiffer *et al.*, 2006 ▸) and non-interferometric ones using, for example, different types of Hartmann masks (Wen *et al.*, 2010 ▸; Olivo *et al.*, 2011 ▸; Zakharova *et al.*, 2018 ▸, 2019*a* ▸,*b* ▸).

In this article, we concentrate on the non-interferometric techniques through Shack–Hartmann (or Hartmann–Shack) wavefront sensors (Mayo & Sexton, 2004 ▸; dos Santos Rolo *et al.*, 2018 ▸; Reich *et al.*, 2018*a* ▸) for hard X-rays (SHSX). Wavefront deformations induced by the object under investigation are analyzed using a regular arrangement of microlenses, each of which creates a reference point. The examination of the deviation of these reference points allows us to determine the deformations of the wavefront.

Section 2 describes designs with cylindrical and parabolic crossed linear lenses using different 3D printing approaches. Three different types of 2D lens arrays were fabricated using a 3D DLW (direct laser writing) technology based on two-photon absorption (Photonic Professional GT2, NanoScribe GmbH). The gain, average spot sizes, aberrations and homogeneity in terms of visibility and gain of the different SHSX designs have been evaluated and are presented in Section 3. A comparison of SHSX prototype performances to investigate a diamond X-ray lens used as a test object can be found in Section 3.4. In Section 4 an interleaving approach is introduced, to overcome the current resolution and fabrication technology limitations of the SHSX, given by the 3D-DLW technique. The image quality and characterization of a diamond lens, using broad-band synchrotron radiation (KARA, KIT, Karlsruhe, Germany), shows the potential of sample-shift multi-contrast X-ray imaging. In Section 5 the degradation of the polymer SHSX originated by radiation damage is analyzed and discussed.

## 2. Design evolution and manufacturing of the Shack–Hartmann wavefront sensors based on 2D refractive lens arrays   

### 2.1. Conception of Shack–Hartmann sensor as a 2D polymer refractive lens array   

Shack–Hartmann sensors (SHS) have been known since the early twentieth century in the visible light range. In 1900, Johannes Hartmann created the first tool to check for approximate focus, and to measure aberrations in the mirrors and lenses of large telescopes (Hartmann, 1900 ▸). This tool, called the Hartmann mask or Hartmann sensor (HS), initially consisted of an opaque screen with numerous holes. Each hole acted as an opening to isolate a small group of light beams, which could be traced to determine any deviation in direction of propagation. This deviation would correspond to the local slope of the wavefront, thus detecting wavefront modifications associated with the quality of the image. Years later, the HS was modified by replacing the apertures by an array of lenslets, thus increasing the signal-to-noise ratio (Shack & Smith, 1971 ▸). Since then, HS and SHS have continuously evolved, and the sensors have also been gaining attraction in the X-ray regime (Mayo & Sexton, 2004 ▸; Reich *et al.*, 2018*a* ▸; Letzel *et al.*, 2019 ▸) in the recent past. dos Santos Rolo and collaborators demonstrated the use of a 2D array of cylindrical polymer refractive lenses as a SHSX. Furthermore, this made fast single-shot multi-contrast imaging of the dynamics of materials with spatial resolution in the micrometre range possible (dos Santos Rolo *et al.*, 2018 ▸). In this section, we will discuss the evolution of approaches to implement SHS in a hard X-ray regime and introduce a new parabolic-shaped lens design, consider the overall influence of the lens shape, and present a way to overcome the former limitations of the 3D DLW technology to increase the sensor field-of-view (FoV).

### 2.2. SHSX design based on continuous hollow cylindrical lenses   

**Figure 1:**
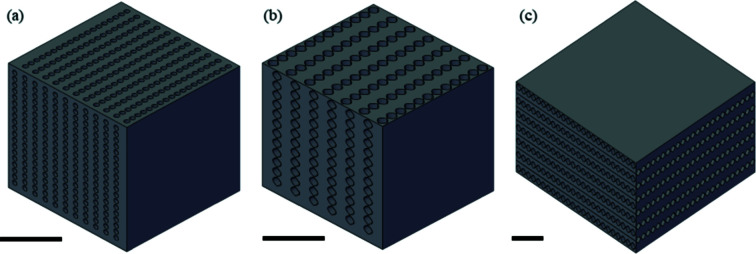
Evolution of SHSX designs: SHSX v1.0 (*a*), SHSX v2.0 (*b*) and SHSX v2.1 (*c*). Sscale bars are 0.5 mm.

The starting pattern developed to study the influence on the lens shape is based on the prototype reported by dos Santos Rolo and collaborators (dos Santos Rolo *et al.*, 2018 ▸). Fig. 1 ▸ shows the SHSX v1.0 design with cylindrical continuous lenses. The 2D focusing lenses are formed by orthogonal oriented cylindrical holes behind each other, where the interception of two perpendicular cylinders is forming a single 2D lens. The holes have diameters of 40 µm. The pitch of the holes in one direction is 50 µm. Since the refracting power of one lens is very low, ten lenses are stacked behind each other. Laterally, this results in an array of 20 × 20 compound refractive lenses (CRL) in an area of 1 mm × 1 mm, which is an improvement of a factor of four compared with the work of dos Santos Rolo *et al.* (2018 ▸). However, this design has some limitations and specific features, in particular the limitation of the FoV to 1 mm × 1 mm and spherical aberration of the lenses. Here we aim to study the effect of the lens shape on the performance of the array, as well as the new approaches to increasing the FoV.

### 2.3. SHSX design based on continuous hollow parabolic lenses   

The SHSX design based on continuous hollow parabolic lenses consists of continuous 1D parabolic cavities with a radius at the parabola apex of 20 µm and a pitch of 170 µm, as shown Fig. 1(*b*) ▸. The overall volume of 1 mm × 1 mm × 1 mm remains, giving a total of 12 × 12 projected spots. A second parabolic shape prototype was developed, striving to increase the FoV. A prototype with a total volume of 2 mm × 2 mm × 1 mm was patterned [Fig. 1(*c*) ▸]. The limitation imposed by the 3D DLW technique is bypassed using a different printing approach: a prototype with a total volume of 2 mm × 2 mm × 1 mm [Fig. 1(*c*) ▸] was explored.

## 3. Characterization of produced arrays   

All X-ray characterization experiments were carried out at the TOPO-TOMO beamline of the KARA synchrotron facility (KIT, Karlsruhe, Germany). Characterization and focal distance measurements of the X-ray lens array were performed using a monochromatic beam with 8.5 keV X-ray energy. The acquisition of radiographic images was performed using the CMOS camera Phantom v2640, lens-coupled to a 50 µm LYSO scintillator. The effective pixel size was 5.3 µm (magnification ∼2.5). One hundred images were taken for each measurement with a frame rate of 100 frames s^−1^ and an exposure time of 700 µs. Before analysis, each set of 100 images was averaged to increase the signal-to-noise ratio.

### 3.1. Average gain and focus definition   

**Figure 2:**
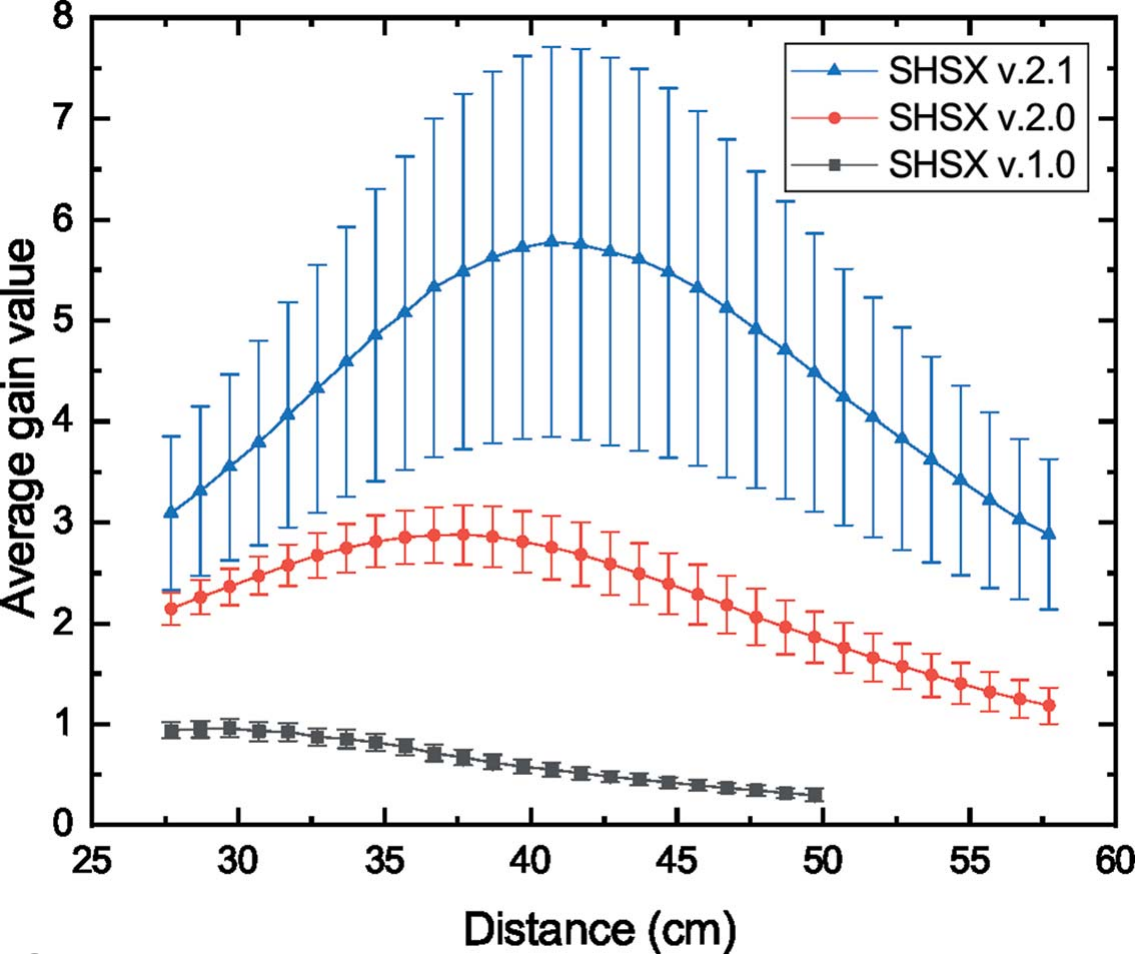
Dependence of average gain values for different generations of SHSX on scanning distance.

The gain of a CRL is defined as the ratio of the on-axis image intensity in the image plane with the lens in place to the corresponding intensity without the lens (Pantell *et al.*, 2001 ▸). The focal length of X-ray refractive lenses can be calculated using the formula (Snigirev *et al.*, 1996 ▸)
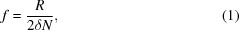
where *R* is the parabola apex radius, *N* is the number of stacked biconcave lenses and δ is the refractive index decrement. The calculated focal lengths for SHSX v1.0, SHSX v2.0 and SHSX v2.1 are 24.6, 41.6 and 41.6 cm, respectively (δ = 3.7 × 10^−6^ and *R* = 20 µm). In this experiment, the focal length was defined as the distance at which the average gain has a maximum value. The experimental focal lengths for SHSX v1.0, SHSX v2.0 and SHSX v2.1 are 29.7 cm, 37.7 cm and 40.7 cm, respectively, as shown in Fig. 2 ▸.

In general, the calculated focal lengths have been reproduced well. The remaining difference between the theoretical and experimental focal distances can be explained by the following factors: the refractive index decrement is calculated theoretically and does not take into account variations in the chemical composition of the commercially available IP-S photoresist; the photoresists used to produce the different generations of SHSX are from different batches; the presence of areas with production defects effectively spreads the individual focal lengths in the array. The same arguments could be related to changes in absolute values of visibility and gain between SHSX v2.0 and v2.1 (discussed in Section 3.3).

### 3.2. Average spot size and astigmatic aberration quantification   

**Figure 3:**
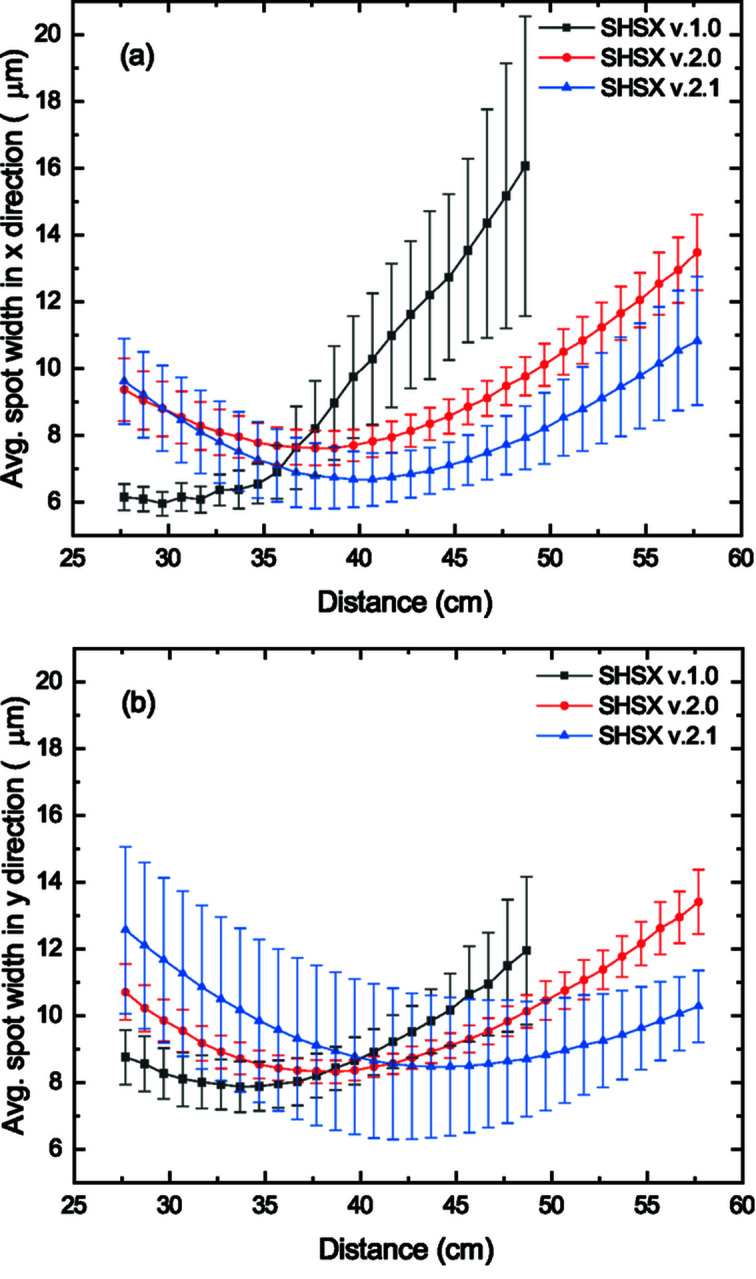
Dependence of average spot width in the *x* (*a*) and *y* (*b*) directions for different generations of SHSX on scanning distance.

According to experimental data [Figs. 3(*a*) and 3(*b*) ▸], the developed lens arrays have astigmatic type aberrations. The focal planes in the *x*- and *y*-directions are located at different distances. Using the relative parameter Δ [equation (2)] (Barannikov *et al.*, 2019 ▸), we can quantify the astigmatic aberrations,
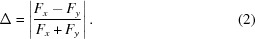
A simple reason for the astigmatic type aberrations is that in the imaging process a finite source at the synchrotron is imaged onto the detector in a caustic, which shortens the horizontal distance to the smallest image size (Reich *et al.*, 2018*a* ▸). A further explanation can originate in the observation that the voxel of the laser used for 3D-DLW does not have spherical symmetry, which results in a difference of lens shapes in the parallel and perpendicular directions to the printing plane. We showed the same effect of printing anisotropy in a previous paper (Mikhaylov *et al.*, 2019 ▸). Barannikov and coauthors (Barannikov *et al.*, 2019 ▸) came to similar conclusions.

Minimizing the size of the focal spots will allow a more sensitive wavefront sampling during interleaving measurements (discussed in Section 4) without beamlet crosstalk through extended tails. The minimum average widths of focal spots for different generations of sensors and distances at which these values were achieved (*F*_*x*_ and *F*_*y*_), as well as the parameter Δ, are presented in Table 1 ▸.

**Figure 4:**
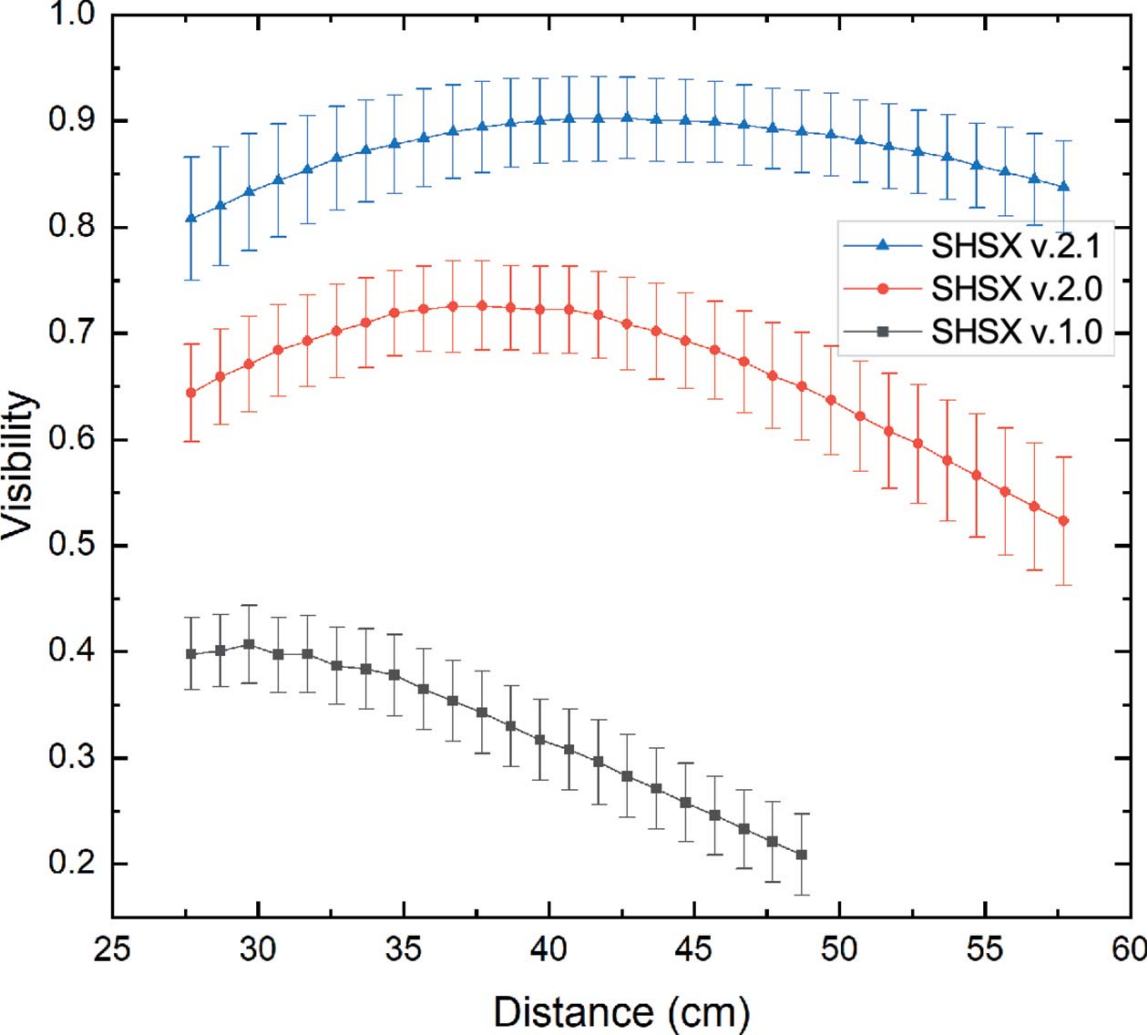
Dependence of average visibility for different generations of SHSX on scanning distance.

Fig. 4 ▸ shows the results of scanning measurements of average visibility [Section 3.3, equation (3)] depending on the distance from the SHSX to the detector. The results indicate that for each generation of SHSX the average value has been improved.

### 3.3. Homogeneity investigation: gain and visibility maps   

**Figure 5:**
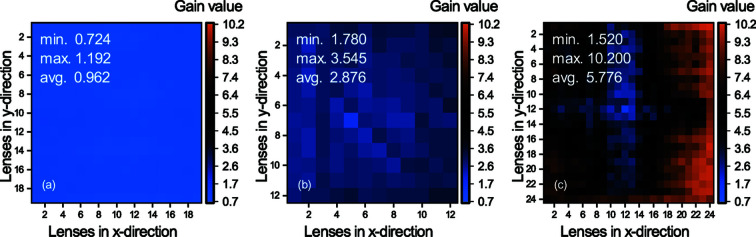
Gain maps of different generations of SHSX at focal distances: SHSX v1.0 (*a*) at 29.7 cm; SHSX v2.0 (*b*) at 37.7 cm; SHSX v2.1 (*c*) at 40.7 cm.

As discussed previously, different generations of SHSX show different performances, which could be caused by structural variations due to different designs or printing defects. Gain and visibility maps are drawn at the focal points to study the homogeneity of the internal structure, qualitative and quantitative evaluation of SHSX performances. Gain maps for the SHSX v1.0 and v2.0 [Figs. 5(*a*) and 5(*b*) ▸] show that the produced structures are relatively homogeneous. The gain of all lenses in these SHSX show uniform values with a standard deviation of 0.089 for v1.0 and 0.293 for v2.0. At the same time, the gain map for the SHSX v1.0 indicates that the sensor absorbs more than it amplifies. This is due to the relatively low X-ray energy and an effective material thickness of up to 1 mm. The gain map for SHSX v2.1 [Fig. 5(*c*) ▸] shows that this sensor has the highest peak gain (10.2) and average gain (5.776). However, this sensor has the largest standard deviation of 1.933. Closer to the center of the sensor, a low gain area (LG area) is detected, which is the region where the individual arrays have been stitched. The area to the right of the LG area will be called the high gain area (HG area).

**Figure 6:**
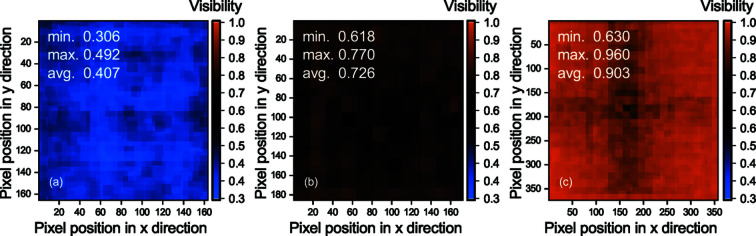
Visibility maps of different generations of SHSX at focal distances: SHSX v1.0 (*a*) at 29.7 cm; SHSX v2.0 (*b*) at 37.7 cm; SHSX v2.1 (*c*) at 40.7 cm.

The visibility has been calculated using equation (3), 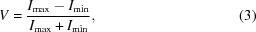
where *I*_max_ is defined as the maximum intensity value and *I*_min_ as the minimum intensity value in the square area of the beamlet zone around each spot. Visibility maps data correlate well with the gain maps. Visibility maps of the sensors SHSX v1.0 [Fig. 6(*a*) ▸] and SHSX v2.0 [Fig. 6(*b*) ▸] show little or no internal structure defects. However, the visibility map for the sensor SHSX v2.1, as well as the gain map for this sensor, indicates the above-mentioned manufacturing artifacts. The nature of internal structural defects can be explained by the appearance of imperfections during the printing process, or these areas have been underdeveloped due to difficult access of chemicals, or as a combination of these factors. However, we think that the main contribution is due to the incomplete development process, as there are regions in SHSX v2.1 with different focal distances.

As a result, we can conclude that the shown SHSX v1.0 acts more like a Hartmann sensor than a Shack–Hartmann. It performs periodic modulations of the wavefront without amplifying the beam intensity at the modulation points [Fig. 6(*a*) ▸]. Since the aim of this work was to develop the Shack–Hartmann sensors for further applications, the SHSX v1.0 was not used for further imaging performance tests. Nevertheless, before it has been shown that SHSX with cylindrical lenses could act like a SHS with a gain of approximately 8 (dos Santos Rolo *et al.*, 2018 ▸).

### 3.4. Multi-contrast imaging performance of SHSX v2.0 and v2.1   

**Figure 7:**
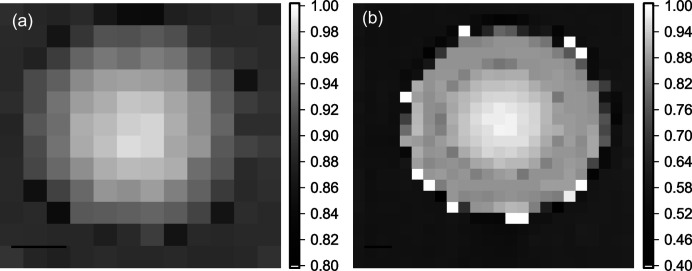
Images of diamond lens in absorption contrast acquired using SHSX v2.0 (*a*) and SHSX v2.1 (*b*). Scale bars are 200 µm.

Tests of imaging performance were carried out using a white beam with a 0.2 mm Al filter. The white filtered beam introduces chromatic aberration, which however is not larger than the imaged source, such that visibility can be largely preserved. As a test object, a diamond parabolic X-ray lens (TISNCM Troisk, Russia) was chosen due to a smooth phase gradient and earlier metrology on it (Gasilov *et al.*, 2017 ▸; dos Santos Rolo *et al.*, 2018 ▸). That object, as a standard test benchmark in our imaging experiments, allows us to compare new data with previous results (Mikhaylov *et al.*, 2019 ▸). The Technological Institute for Superhard and Novel Carbon Materials (TISNCM) in Troitsk, Russia, manufactured the diamond parabolic X-ray lens for this and previous experiments. Nominal dimensions of the diamond lens are: radius of parabola apex, *R* = 200 µm; geometrical aperture, *A* = 900 µm; thickness, *H* = 500 µm (Gasilov *et al.*, 2017 ▸). The diamond lens (DL) has been placed in between the SHSX and the detector. The distance SHSX to DL was 86.5 cm and DL to the detector was 36.7 cm. The location of the SHSX has been chosen as near to the focal point of SHSX v2.0 at 15 keV. In Fig. 7 ▸ an example of imaging in absorption-contrast is shown. Phase-contrast and diffraction-contrast pictures can be found in the supporting information to this article. The lens pitch of SHSX defines the pixel size of imaging. In this experiment, the pixel size of imaging in all types of contrasts is 85 µm. The multi-contrast retrieval was performed by Gaussian beamlet fitting (Reich *et al.*, 2018*a* ▸). The angular resolution in differential-phase contrast mode using SHSX v2.0 is 0.4 µrad, and using SHSX v2.1 is 0.29 µrad (determined as the standard deviation in the undisturbed area).

## 4. Super-resolution multi-contrast imaging of diamond lens by interleaving measurement   

**Figure 8:**
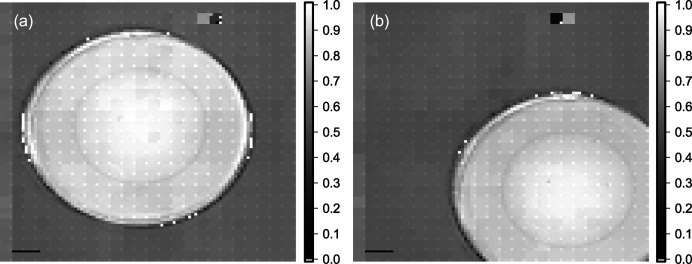
Super-resolution images of diamond lens in absorption contrast acquired using SHSX v2.1: LG area (*a*) and HG area (*b*) of the SHXS v2.1. Scale bars are 200 µm.

To increase the limited spatial resolution, an interleaving measurement was performed. As the beamlet size at the position of the sample is much smaller than the beamlet zone, the beamlets locally only probe a small area of the sample. The sample was measured interleaved with sub-pitch shifts of it. The spatial resolution is defined by the SHSX pitch, the amount of interleaving and beamlet size at the sample position. With the fourfold interleaving, we obtained a nominal spatial resolution of 21 µm. These sample-shift X-ray imaging experiments were also carried out at the KARA synchrotron facility (KIT, Karlsruhe, Germany) using a white beam (Mikhaylov *et al.*, 2019 ▸). Acquisition of radiographic images was performed using a CMOS camera PCO.dimax with a 50 µm-thick LuAg:Ce scintillator. The effective pixel size with the lens optics is 7.3 µm (magnification ∼1.5). The distance between DL and the detector was 16 cm, and the SHSX to DL distance was 91 cm. This configuration has been chosen to achieve a suitable size of focusing spots on the DL and to be able to perform interleaving four times in the *X*- and *Y*-directions. For each measurement 100 images were taken with a frame rate of 0.100 Hz and an exposure time per frame of 1 ms. Before analysis, each set of 100 images was averaged to increase the signal-to-noise ratio. Fig. 8 ▸ shows super-resolution images of a diamond lens in absorption contrast using HG and LG areas of SHSX v2.1. The angular resolution in differential-phase contrast mode is 0.6 µrad (standard deviation of undisturbed area). In this experiment, the angular resolution is lower than in the experiment shown in Section 3 since the distance between DL and the detector is smaller, and the physical and effective pixel sizes of the used detector are bigger. Dark-field pictures can be found in the supporting information to this article.

**Figure 9:**
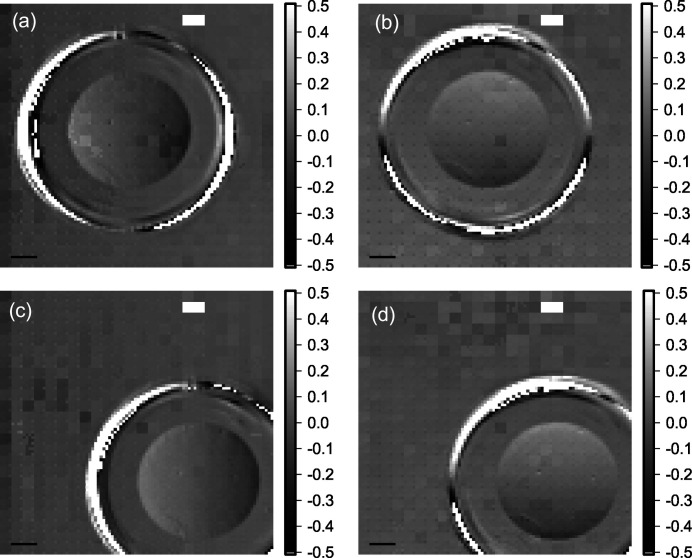
Super-resolution images of diamond lens in differential phase contrast acquired using SHSX v2.1: LG area (*a*, *b*) and HG area (*c*, *d*) of the SHXS v2.1. Scale bars are 200 µm.

The differential phase contrast in all images (Figs. 9 ▸ and 10 ▸) reproduces the gradient in phase shift across the lens, which should be almost linear for a parabolic lens shape. The resolution as inspected by eye is as high as the oversampling with sharp edges at the rims of the lens. Placing the lens in the center of the SHXS v2.1 produces more erroneous patches, which comprise a full 4 × 4 interleaved area. Thus, the error in phase shift can be ascribed to a single faulty lenslet. Nevertheless, it is shown that interleaving takes advantage of the small locally probed area of each beamlet on the sample and thus can gain part of the resolution loss due to sampling one beamlet by several detector pixels.

## 5. Degradation of polymer lens arrays under continuous X-ray illumination   

**Figure 11:**
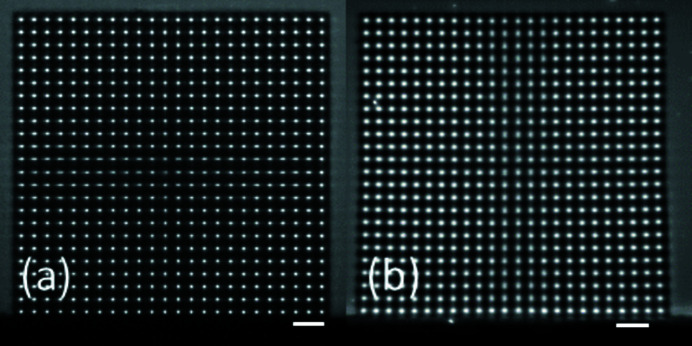
Images of the SHSX v2.1 spot pattern in white beam at the beginning (*a*) and the end of long-time exposure (*b*). Scale bars are 200 µm.

To determine the durability of the SHSX under continuous X-ray irradiation, long-exposure experiments were performed. For usage of the SHSX as regular optical devices in X-ray beamlines a certain durability is of interest. After an exposure of around 15 h, visible shape changes can be observed as shown in Fig. 11 ▸. The size of the SHSX decreased leading to a reduced lens periodicity. This effect of negative photoresist shrinkage is well known to the scientific community (Kunka *et al.*, 2014 ▸; Koch, 2017 ▸).

**Figure 12:**
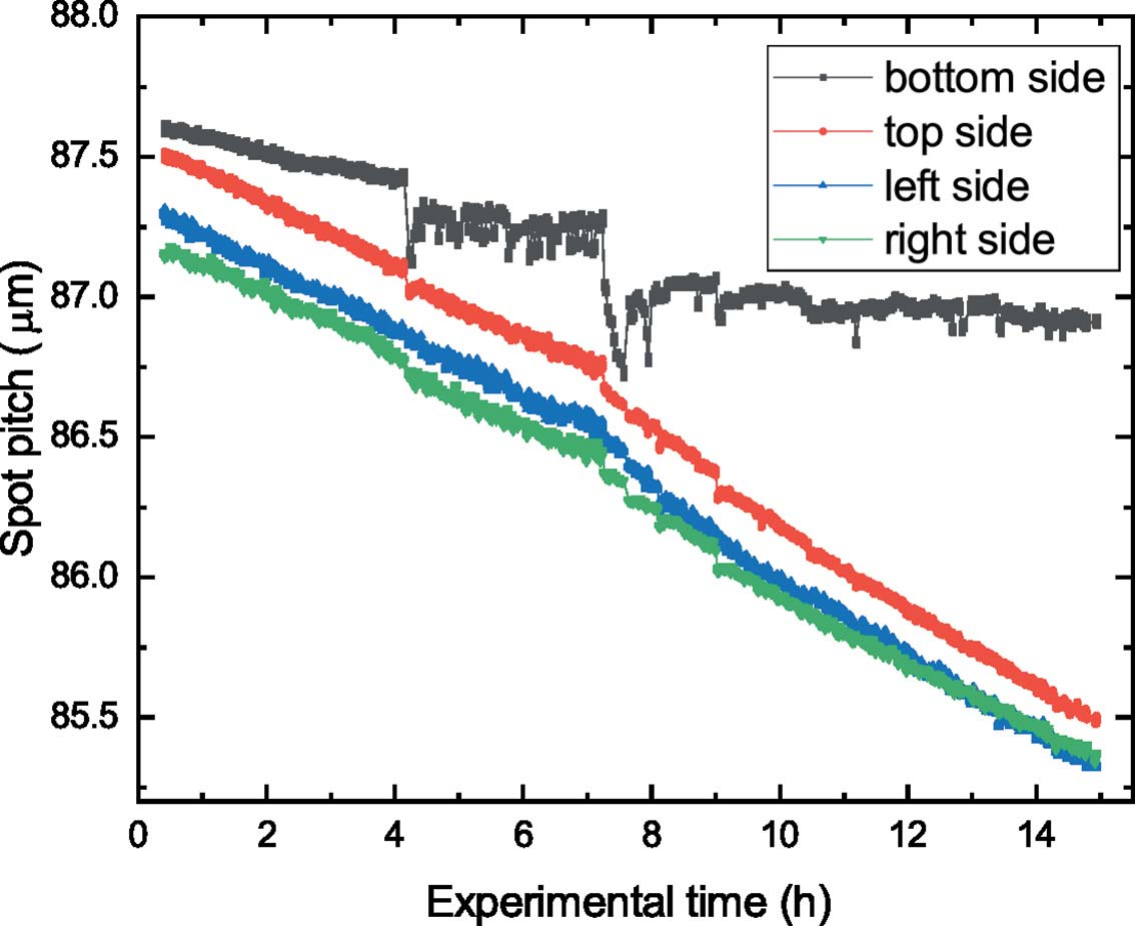
Dependence of the spot pitch of SHSX v2.1 on the experimental time in hours.

To determine the shrinkage in more detail, the average spot pitch is shown in Fig. 12 ▸ for the four SHSX sides. At the beginning, the SHSX had a comparable good rectangular shape with a pitch of around 87.5 µm. With time, the pitches decrease for all four sides. Apart from the bottom side, all sides have shrunken similarly. The bottom side, where the SHSX was mounted on a holder by gluing, showed less shrinkage. This indicates that the mechanical stability was reinforced due to the external rigid holder. The increased shrinkage rate after half the time is attributed to a higher X-ray flux caused by an increased synchrotron ring current.

**Figure 13:**
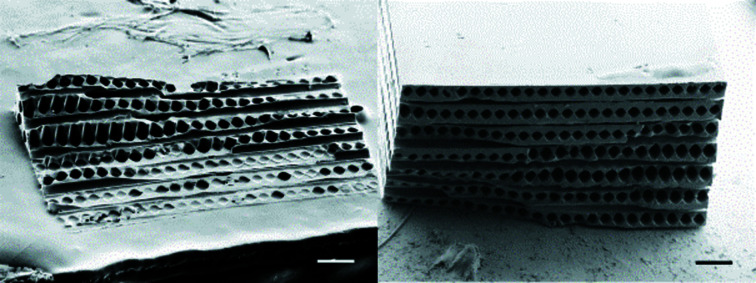
SEM images of the break site of SHSX v2.1. Scale bars are 100 µm.

Furthermore, the mechanical stress caused by the fixation of the bottom side of the SHSX v2.1 to the holder led to the breakage during its separation from it. This stems from buildup of internal strain due to the fixed bottom part relative to the freely shrinking upper part. Fig. 13 ▸ shows an SEM microphotograph of the broken plane parallel to the attachment on the holder. Such strain buildup may be reduced by a different mounting scheme, such as single-point attachment. Nevertheless, such a shrinking should be considered for data evaluation. Inhomogeneous shrinking may affect data analysis, in particular if a Fourier approach is used (Wen *et al.*, 2010 ▸).

**Figure 14:**
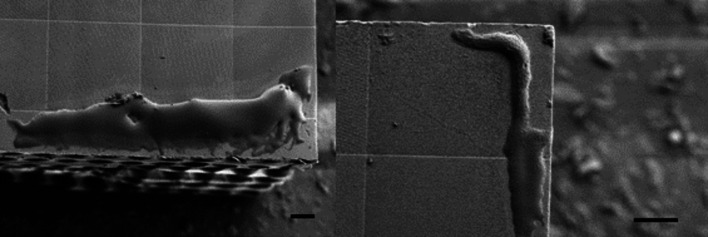
Influxes of unclear nature on the surface of SHSX v2.1 after the last performed experiment. Scale bars are 50 µm.

Systematic investigation of the behavior of the IP-S resist in the X-ray beams, and the study of the radiation resistance are aims of our future work. We have employed here Gaussian fitting procedure for data processing (Reich *et al.*, 2018*b* ▸), so that changes in the periodicity and lateral dimensions of the array are not so critical. Furthermore, the appearance of an influx of unclear nature was noticed (Fig. 14 ▸), which might stems from unreacted chemicals.

## 6. Conclusions   

In the present paper we discussed the evolution and different designs of Shack–Hartmann (Hartmann–Shack) sensors for hard X-rays. Our experiments showed that SHSX based on 2D refractive lens arrays could be used for fast wavefront monitoring and single-shot multi-contrast imaging. We were able to reach an angular resolution of approximately 0.29 µrad. Even in the case of using a sample-shift technique by interleaving measurements at shifted sample positions to increase the spatial resolution, the measurement time spend on measurements is still in a range of seconds, which is much less than in case of using other wavefront-sensitive instruments. The spatial resolution in these experiments was demonstrated to be 21 µm. Chromatic aberrations in white-beam illumination, despite possible concerns are not detrimental to the measurement, but only reduce sensitivity gradually. Nevertheless, we noticed strong effects of radiation damage. The SHSX shrinks in size within 15 h under hard X-ray white beam illumination. In the case of using a two-dimensional Gaussian function fit procedure instead of Fourier extraction to analyze the spot pattern, the small shrinkage observed was not critical.

## Supplementary Material

Figure S1: Images of a diamond lens in differential phase contrast; Fig. S2: Images of diamond lens in dark-field contrast; Fig. S3: Super-resolution images of diamond lens in dark-field contrast. DOI: 10.1107/S1600577520002830/ve5120sup1.pdf

## Figures and Tables

**Figure 10:**
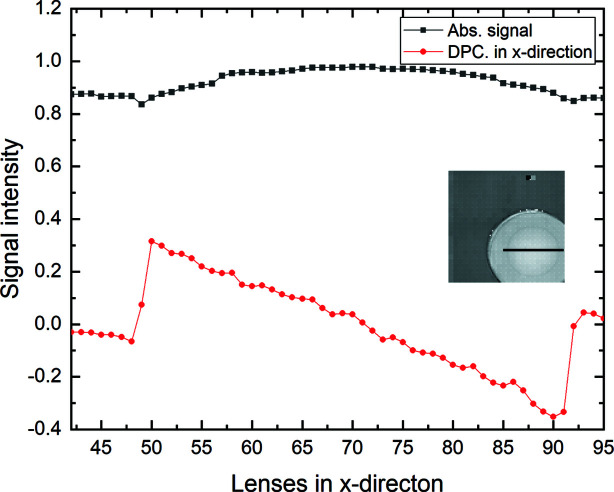
Comparison of normalized differential phase and absorption signals [Figs. 8(*b*) ▸ and 9(*c*) ▸] along shown lines for SHSX v2.1.in the HG area

**Table 1:** Minimal average width of focal spots at given distances and the astigmatism parameter Δ

Sensor generation	Direction	Minimum width (µm)	Distance (cm)	Δ (%)
SHSX v1.0	*F*_*x*_	5.9 ± 0.4	29.7	6.31
	*F*_*y*_	7.9 ± 0.8	33.7	
SHSX v2.0	*F*_*x*_	7.6 ± 0.5	37.7	1.31
	*F*_*y*_	8.3 ± 0.3	38.7	
SHSX v2.1	*F*_*x*_	6.7 ± 0.7	41.7	2.34
	*F*_*y*_	8.7 ± 2.1	43.7	
