# Supplementary material for: Shack–Hartmann wavefront sensors based on 2D refractive lens arrays and super-resolution multi-contrast X-ray imaging
Source: J Synchrotron Radiat. 2020 Apr 22;27(Pt 3):788–95. doi: 10.1107/S1600577520002830 (PMC7206553; doi:10.1107/S1600577520002830)
Supplement: Supplementary file 1 [file s-27-00788-sup1.pdf]

JOURNAL OF SYNCHROTRON RADIATION

Volume 27 (2020)

Supporting information for article:

Shack–Hartmann wavefront sensors based on 2D refractive lens arrays and super-resolution multi-contrast X-ray imaging

Andrey Mikhaylov, Stefan Reich, Margarita Zakharova, Vitor Vlnieska, Roman Laptev, Anton Plech and Danays Kunka

S1. Multi-contrast imaging performance of SHSX v2.0 and v2.1. Phase-contrast and dark-field contrast images for *Section 3.4*

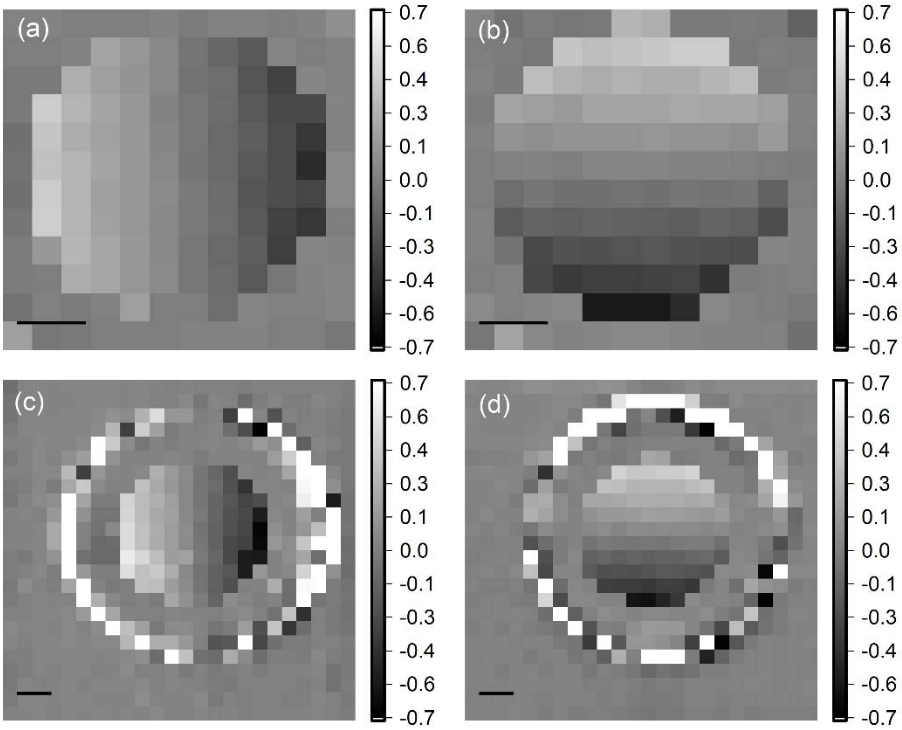

Figure S1 Images of diamond lens in differential phase contrast acquired using SHSX v2.0 (a, b) and SHSX v2.1 (c, d) (scale bars are 200 μm).

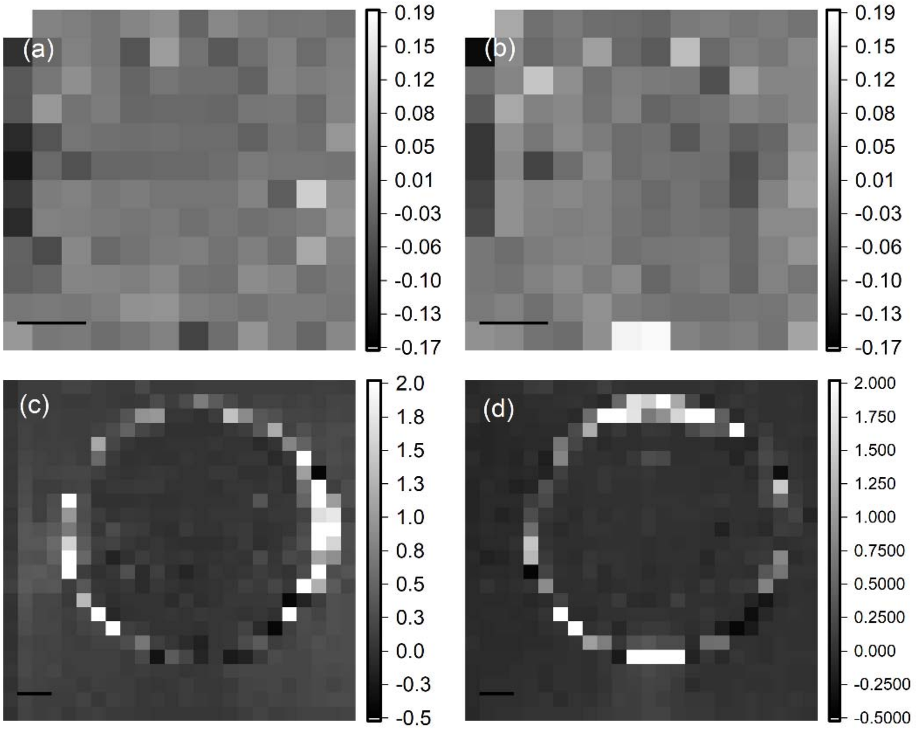

Figure S2 Images of diamond lens in dark-field contrast acquired using SHSX v2.0 (a, b) and SHSX v2.1 (c, d) (scale bars are 200 μm).

S2. Super-resolution multi-contrast imaging of diamond lens by interleaving measurement. Dark-field pictures for *Section 5*.

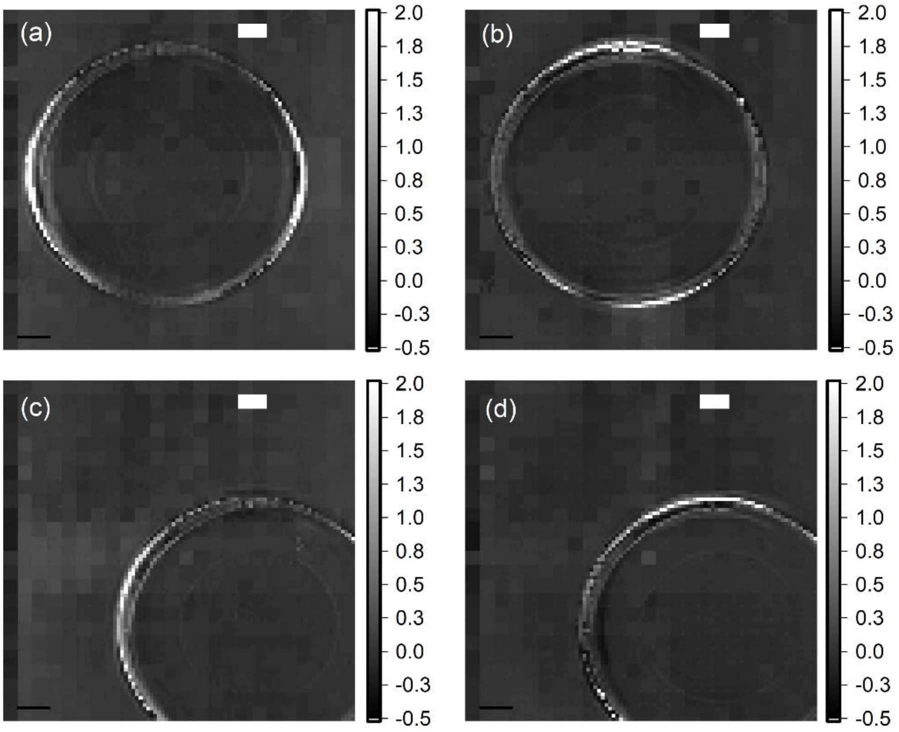

Figure S3 Super-resolution images of diamond lens in dark-field contrast acquired using SHSX v2.1: LG area (a, b) and HG area (c, d) of the SHXS v.2.1 (scale bars are 200 µm).
